# Symptomatological Transversality and the Absence of Pathognomonic Symptoms in Psychiatry

**DOI:** 10.1192/j.eurpsy.2023.2121

**Published:** 2023-07-19

**Authors:** A. I. Gomes, S. Jesus, G. Simões, S. Vicente

**Affiliations:** Centro Hospitalar do Baixo Vouga EPE, Aveiro, Portugal

## Abstract

**Introduction:**

The diagnosis of the main psychiatric syndromes is still almost exclusively phenotypic and depends essentially of the recognition of characteristic signs and symptoms. The clinical evalution allows the formulation of a set of differential diagnoses, according to the pathological meaning of certain symptomatic patterns and combinations. Aside from the entire dependence on the clinical interview, there are still no complementary psychiatric diagnostic exams and it is also worth noting the absence of pathognomonic symptoms.

**Objectives:**

Through the presentation of the case of a patient with Bipolar Affective Disorder who manifests, during a manic episode, a Capgras delusion, we intend to approach the heterogeneity of the manifestation of some symptoms that tend to be specific of concrete psychiatric syndromes.

**Methods:**

Clinical case presentation and non-systematic literature review using Pubmed plataform.

**Results:**

AB, female, 49 years old, diagnosed with Bipolar Affective Disorder. Hospitalized for a manic episode with dysphoric mood, increased energy levels and delusional activity of grandiose and persecutory content. During hospitalization, a Capgras delusion centered on the husband emerged: he was replaced by a stranger, I was able to detect him by smell.

Capgras delusion is a delusional misidentification syndrome characterized by the belief that someone close has been replaced by an imposter. Despite being a rare syndrome, vastly more common in schizophrenia, affecting about 73% of cases, it can also occur in other psychiatric conditions such as dementia syndromes and, less often, mood disorders (16.7%).

Additionally, there are several examples that demonstrate the versatility of psychiatric symptom occurrence in different diagnoses, with first-rank symptoms serving as an example. Described in 1959 by Kurt Schneider, they were considered specific symptoms of schizophrenia, assuming this diagnosis based on the recognition of only one symptom. Over time, its pathognomonic character has become extinct, and its detection in mood disorders and acute psychotic disorder is relatively common.

Another example is the overlap between depressive and anxious symptoms. In fact, anxiety symptoms occur in about 85% of patients diagnosed with depressive disorder and, in turn, the presence of depressive symptoms in about 90% of patients diagnosed with anxiety disorder. This evidence has allowed, over time, a review of the diagnostic criteria for these disorders, leading to a progressive blurring of the threshold between them.

**Conclusions:**

Psychiatric diagnosis is still a delicate task, totally dependent on the clinical interview. The lack of analytical and imaging tests, as well as the absence of pathognomonic symptoms, constitute a particular challenge in diagnosis. For this reason, we highlight the importance of recognizing combinations and patterns of symptoms rather than the specificity of just one symptom.

**Disclosure of Interest:**

None Declared

